# Novel In Situ Growth of ZIF-8 in Porous Epoxy Matrix for Mechanically Robust Composite Electrolyte of High-Performance, Long-Life Lithium Metal Batteries

**DOI:** 10.3390/molecules27217488

**Published:** 2022-11-03

**Authors:** Wenjie Zhang, Jianlin Long, Haijun Wang, Jinle Lan, Yunhua Yu, Xiaoping Yang

**Affiliations:** 1 State Key Laboratory of Organic-Inorganic Composites, College of Materials Science and Engineering, Beijing University of Chemical Technology, North Third Ring Road 15, Chaoyang District, Beijing 100029, China; 2 State Key Laboratory of Fluorinated Functional Membrane Materials, Zibo 256401, China; 3 Foshan (Southern China) Institute for New Materials, Foshan 528200, China

**Keywords:** lithium metal batteries, polymer electrolyte, in situ growth, ZIF-8, thermal stability, lithium dendrites

## Abstract

Polymer electrolytes (PEs) with high flexibility, low cost, and excellent interface compatibility have been considered as an ideal substitute for traditional liquid electrolytes for high safety lithium metal batteries (LMBs). Nevertheless, the mechanical strength of PEs is generally poor to prevent the growth of lithium dendrites during the charge/discharge process, which seriously restricts their wide practical applications. Herein, a mechanical robust ZIF-8/epoxy composite electrolyte with unique pore structure was prepared, which effectively inhibited the growth of lithium dendrites. Meanwhile, the in situ growth of ZIF-8 in porous epoxy matrix can promote the uniform flux and fast transport of lithium ions. Ultimately, the optimal electrolyte shows high ionic conductivity (2.2 × 10^−3^ S cm^−1^), wide electrochemical window (5 V), and a large Li^+^ transference number (0.70) at room temperature. The Li||NCM811 cell using the optimal electrolyte exhibits high capacity and excellent cycling performance (83.2% capacity retention with 172.1 mA h g^−1^ capacity retained after 200 cycles at 0.2 C). These results indicate that the ZIF-8/epoxy composite electrolyte is of great promise for the application in LMBs.

## 1. Introduction

Lithium metal is considered an extremely promising anode material because of its high theoretical capacity (3860 mA h g^−1^) and the lowest electrochemical potential (−3.040 V vs. the standard hydrogen electrode) [1,2,3,4]. However, the high reactivity of lithium metal anodes is prone to continuous side reactions with organic liquid electrolytes [5,6], and the uncontrolled growth of lithium dendrites and the formation of dead lithium [7,8,9] cause a severe safety hazard. Therefore, numerous studies have optimized liquid electrolytes by adjusting the electrolyte composition, upgrading the lithium salt concentration or adding functional additives [10] to form a protective layer on the lithium anode surface and stabilize the solid electrolyte interface (SEI) [11,12]. Nevertheless, as these additives continue to be consumed, their inhibitory effect on Li dendrite growth decreases, and the problems of leakage, flammability and volatilization of traditional liquid electrolytes are still difficult to solve [13,14].

The solid electrolyte replaces the liquid electrolyte, which can avoid the risk of liquid leakage and improve the safety performance of the battery [15,16,17]. Solid-state electrolytes include inorganic ceramic electrolytes and solid polymer electrolytes (SPEs) [18,19]. Compared with brittle inorganic ceramic electrolytes with large interfacial resistance between solid electrolytes and electrodes [20], SPEs have been widely studied due to their advantages of excellent interfacial compatibility, high ionic conductivity, light weight, low cost, and high flexibility [21,22,23]. Meanwhile, the ideal polymer matrix should have sufficient mechanical strength, good thermal and structural stability, and be able to play the role of skeletal support [18,24]. Previous studies [25,26,27] have found that epoxy resin has the advantages of great structural designability, low cost, and high mechanical strength, which can be used as a promising candidate material for polymer electrolyte matrix. In addition, polymer-based electrolyte systems usually need to be modified to further improve the mechanical strength and electrochemical performance [28,29]. The most common method is to add fillers in the system [30]. Compared with traditional inorganic fillers, metal-organic frameworks (MOFs) have the advantages of easy synthesis, high porosity, large specific surface area, and excellent mechanical and thermal stability [31,32,33].

As a subset of MOFs, ZIF-8 not only has the advantages of MOFs, but also has better chemical and thermal stability and a low processing cost [34,35], which makes it widely concerned in the field of high-energy LMBs. Hence, many ZIF-8/polymer composite electrolytes have been studied [36,37], which have good inhibitory effect on the growth of Li dendrites. However, the polarization test of Li deposition/stripping at relatively low current densities (0.1–0.5 mA cm^−2^) with low areal capacities (0.1–1 mA h cm^−2^) [36,38,39] cannot meet the requirements for high specific energy LMBs. Therefore, it is necessary to develop a new type of composite electrolyte [40,41,42] with simple preparation process, high ionic conductivity, good contact with electrode interface, and effective inhibition of lithium dendrite growth even at high current density.

Here, we design a convenient method to in situ grow ZIF-8 in a unique pore channel of porous epoxy resin membrane to prepare a ZIF-8/epoxy composite electrolyte. The composite membrane has the advantages of high mechanical strength, good thermal stability, and chemical stability. Meanwhile, the composite electrolyte membrane with unique pore channel has high electrolyte uptake (566.7%), good room temperature ionic conductivity (2.2 × 10^−3^ S/cm), and wide electrochemical window (5 V). In the polarization test of Li||Li symmetrical cell, the prepared PEME-3:7/ZIF-8(15%) exhibits a steady Li plating/stripping behavior, even at a high current density of 1.5 mA cm^−2^ for 6 mA h cm^−2^. Moreover, the assembled Li||PEME-3:7/ZIF-8(15%)||LiFePO_4_ and Li||PEME-3:7/ZIF-8(15%)||NCM811 batteries deliver favorable rate performance and cycle performance. Therefore, a dendritic-free LMB with simple process, excellent electrochemical performance, and good cycle stability has been successfully prepared in this work.

## 2. Results and Discussion

**Figure 1:**
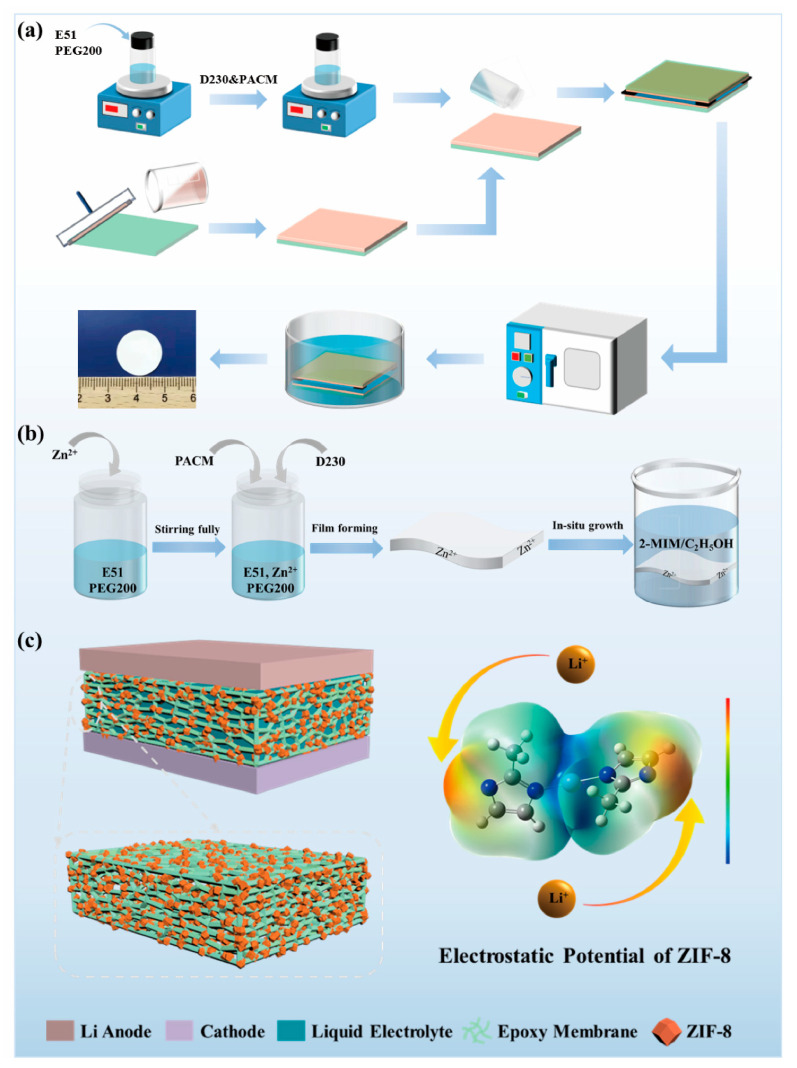
Schematic diagram of (**a**) the preparation of PEME-x membranes; (**b**) the preparation of PEME-3:7/ZIF-8 composite membranes, and (**c**) Li metal battery with PEME-3:7/ZIF-8 electrolyte and electrostatic potential of ZIF-8.

To achieve a polymer electrolyte with good performance for LMBs, a porous epoxy resin matrix electrolyte (PEME-3:7) with optimized morphologies and properties was prepared by compounding the curing agents (soft D230 and rigid PACM) and regulating their ratios (Figure 1a). Furthermore, to further enhance the electrochemical performance of the PEME-3:7, we designed a novel method to in situ grow ZIF-8 on the surface and pore surfaces of the PEME-3:7 membrane to obtain PEME-3:7/ZIF-8 composite electrolytes (Figure 1b,c). The electronegativity and nucleophilicity of ZIF-8 endow the PEME-3:7/ZIF-8 electrolyte with excellent electrochemical performance.

### 2.1. Optimization of Porous Epoxy Resin Matrix

**Figure 2:**
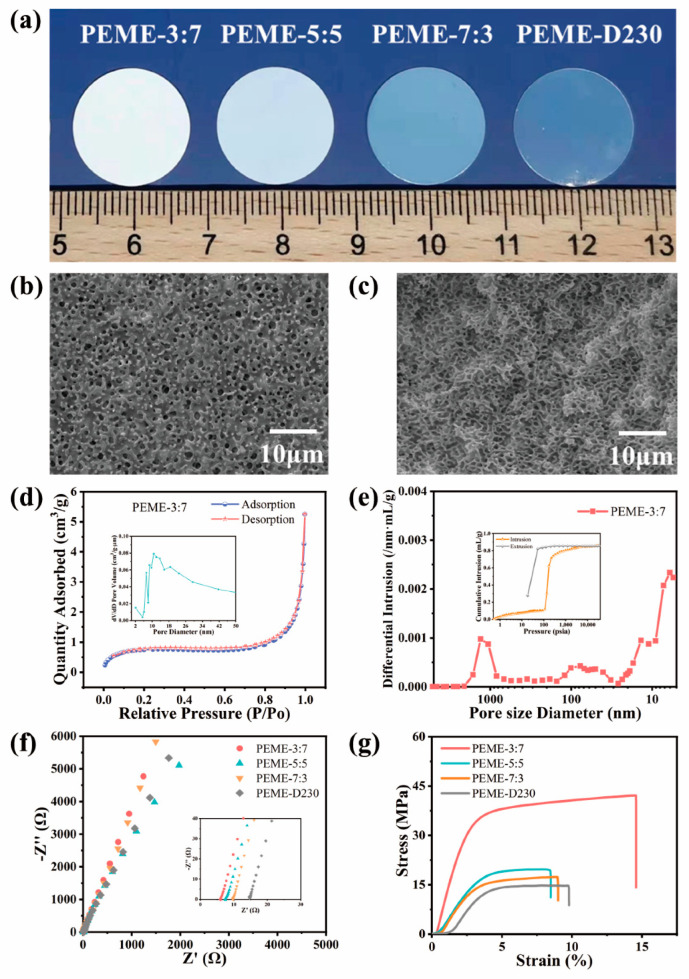
(**a**) Digital photos of PEME-x membranes. The (**b**) surface and (**c**) cross-section SEM images of PEME-3:7. (**d**) N_2_ adsorption-desorption isotherm of PEME-3:7 (inset: the pore size distribution of the membrane). (**e**) Pore size distribution of PEME-3:7 membrane under mercury intrusion test (inset: the mercury intrusion-evolution curve of the membrane). (**f**) Impedance spectrum of SS||PEME-x||SS cells at room temperature. (**g**) The tensile stress-strain curves of the PEME-x membranes.

The epoxy resin membranes should have excellent mechanical strength, high electrolyte uptake, appropriate crosslinking density and pore size to facilitate the swelling of the membranes [25,26,43]. In this work, epoxy resin E51 was selected as the polymer matrix, PEG200 as the porogen agent, and PACM and D230 as the curing agent compounded to control the pore structure by regulating their ratios and to obtain good electrochemical properties and high mechanical strength. On increasing the proportion of D230 in the system, the transparency of the epoxy resin membranes gradually increased, changing from white opaque to transparent (Figure 2a). Undoubtedly, this color change is mainly attributed to the change in the microstructure of the membranes after adding different ratios of curing agents, which affects the refractive index. As shown in Figure 2b,c and Figure S1, the surface and cross-section of PEME-3:7 membrane present continuous and pore structure with uniform pore size distribution. As the proportion of D230 continues to increase, the pore size gradually decreases, which is consistent with the results of the BET test (Figure S2). As shown in Figure 2d and Figure S2, the mesoporous pore size distribution of the PEME-x membranes is mostly between 17 nm and 35 nm, and the average mesoporous pore size of the PEME-3:7 membrane is about 24 nm. The macroporous pore size of the PEME-3:7 membrane obtained by mercury intrusion test is 300 nm (Figure 2e). The continuous penetrating mesopore–macropore coexistence structure of the PEME-3:7 membrane tends to facilitate the electrolyte absorption, which in turn enhances the ionic conductivity. As shown in Figure S3, the electrolyte uptake of PEME-3:7 membrane (366.1%) is higher than that of other electrolyte membranes. Therefore, the ionic conductivity of PEME-3:7 is also superior to other electrolytes (Figure 2f and Figure S3). Furthermore, the tensile strength and Young’s modulus of the membranes decrease with the addition of D230, as shown in Figure 2g and Figure S4, the PEME-3:7 membrane has the best mechanical properties. After comprehensive analysis, the PEME-3:7 was selected as the epoxy resin matrix of the composite electrolyte membrane.

### 2.2. Design of ZIF-8/Epoxy Composite Electrolyte

**Figure 3:**
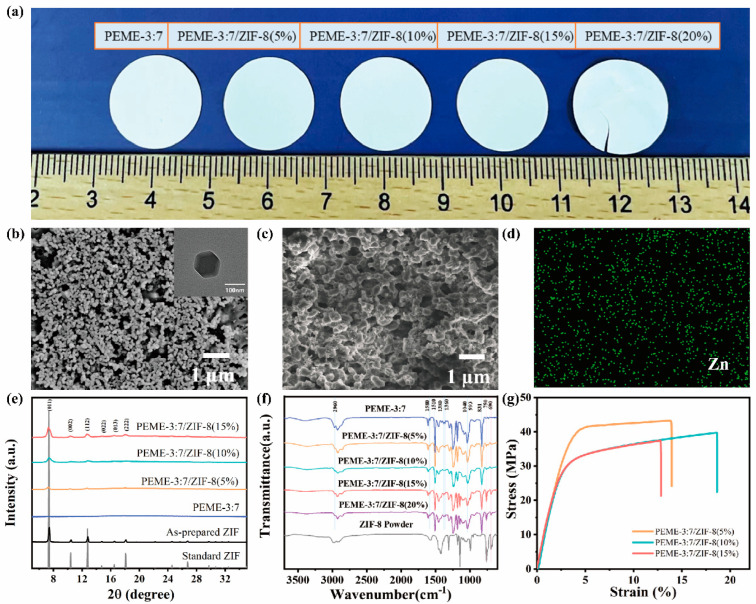
(**a**) Photographs of epoxy resin membranes with different ZIF-8 contents. The SEM images of PEME-3:7/ZIF-8(15%); (**b**) surface (inset: the corresponding TEM image of ZIF-8), and (**c**) cross-section; (**d**) Zn EDS mapping of PEME-3:7/ZIF-8(15%) membrane; (**e**) XRD patterns; (**f**) FTIR spectra, and (**g**) tensile stress–strain curves of different components.

Figure 3a shows the intuitive photographs of the PEME-3:7/ZIF-8 membranes with different ZIF-8 contents. The PEME-3:7/ZIF-8 membranes are all white and opaque (Figure 3a). ZIF-8 are successfully grown in situ on the surface and the internal pore surface of the PEME-3:7 membranes (Figure 3b,c and Figure S5). Moreover, with the increase of the mass fraction of Zn(CH_3_COO)_2_·2H_2_O, the growth amount of ZIF-8 is increased gradually. However, the PEME-3:7/ZIF-8(20%) membrane becomes very fragile due to the overgrowth of ZIF-8, making it difficult for practical application (Figure 3a and Figure S5e,f). The ZIF-8 particles in PEME-3:7/ZIF-8(15%) have a uniform particle size of about 100 nm with a rhombic dodecahedral shape (Figure 3b). Figure 3d and Figure S6 display the SEM and Zn EDS mapping results of the PEME-3:7/ZIF-8 membranes, where the uniform distribution of ZIF-8 can be clearly observed.

The growth of ZIF-8 in the PEME-3:7/ZIF-8 membranes were investigated using XRD. The PEME-3:7/ZIF-8 membrane exhibits the same XRD sharp peaks with the standard ZIF-8 and the as-prepared ZIF-8 powder. The crystal planes of the (011), (002), (112), (022), (013), and (222) facets show peaks at 7.3°, 10.3°, 12.6°, 14.6°, 16.4°, and 17.9° [44], respectively (Figure 3e). For further proving the presence of ZIF-8 in the composite membranes, the membranes were characterized using FTIR. The FTIR spectra of the PEME-3:7, the PEME-3:7/ZIF-8(5~20%), and the ZIF-8 powder are listed in Figure 3f. The presence of the adsorption of ZIF-8, such as C-H (690 cm^−1^, 993 cm^−1^) and N-H (750 cm^−1^) bands in the spectra of PEME-3:7/ZIF-8 membranes confirms the successful in situ growth of ZIF-8. Moreover, no obvious changes related to the absorption peaks of the C-H (2960 cm^−1^) in -CH_3_, C-O-C (1040 cm^−1^), bis-methyl (1360 cm^−1^, 1380 cm^−1^), or benzene ring (831 cm^−1^, 1510 cm^−1^, 1580 cm^−1^) of the epoxy are observed after in situ growth of ZIF-8.

In order to investigate the porous structure of the PEME-3:7/ZIF-8 membranes, the BET and automated mercury intrusion tests were performed. As shown in Figure S7a–c, the average mesopore diameters of PEME-3:7/ZIF-8(5%), PEME-3:7/ZIF-8(10%), PEME-3:7/ZIF-8(15%) are 40 nm, 33 nm, and 20 nm, respectively. As shown in Figure S7d, the average macropore diameter of PEME-3:7/ZIF-8(15%) is 367 nm. Compared with the pure PEME-3:7 membrane (Figure 2d,e), the pore size is slightly different, indicating that the in situ growth of ZIF-8 has an effect on the pore structure, but the mesopore–macropore coexistence structure is still maintained.

Furthermore, Figure 3g presents the tensile stress–strain curves of the PEME-3:7/ZIF-8 membranes. Apparently, the PEME-3:7/ZIF-8 membranes maintain the excellent mechanical properties of the PEME-3:7 membrane with high tensile strength and Young’s modulus, which is promising to inhibit the growth of lithium dendrites. The tensile strength and Young’s modulus of the PEME-3:7/ZIF-8(15%) are 37.4 MPa and 1154.9 MPa, respectively. As shown in Figure S8, the PEME-3:7/ZIF-8(15%) membrane remains flexible and free-standing even after the activation of the liquid electrolyte.

**Figure 4:**
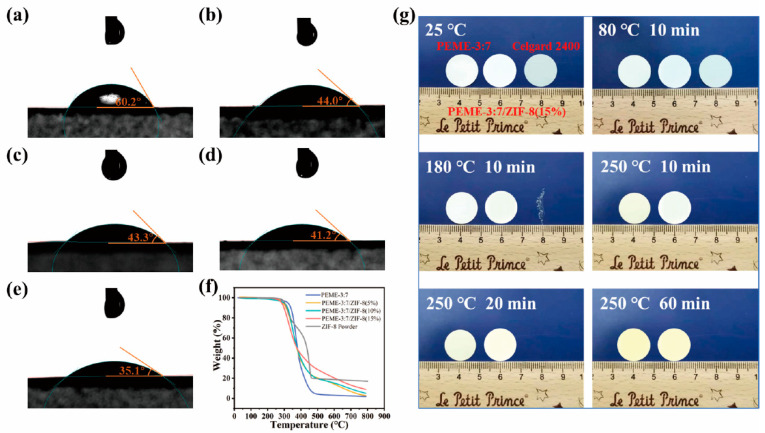
Surface contact angles of (**a**) Celgard 2400; (**b**) PEME-3:7; (**c**) PEME-3:7/ZIF-8(5%); (**d**) PEME-3:7/ZIF-8(10%), and (**e**) PEME-3:7/ZIF-8(15%) with 1 M LiPF_6_/EC: DME (1:1, *v*/*v*); (**f**) TGA test curves of different components, and (**g**) Schematic changes in appearance of Celgard 2400, PEME-3:7, and PEME-3:7/ZIF-8(15%) membranes with temperature and time.

The surface infiltration ability and wetting behavior of the membranes were investigated by contact angle measurements. As shown in Figure 4a–e, Celgard 2400 exhibits a poor wetting performance for liquid electrolytes with a large contacting angle of 60.2°. In contrast, the contact angles of PEME-3:7/ZIF-8(0~15%) are 44.0°, 43.3°, 41.2°, and 35.1°, respectively. The remarkable infiltration behavior of PEME-3:7/ZIF-8 are achieved by the synergistic effect of the surface polarity of PEME-3:7 and high porosity of ZIF-8. The excellent electrolyte infiltration performance is conducive to improving the electrochemical properties of PEME-3:7/ZIF-8. As shown in the TGA curves (Figure 4f), the PEME-3:7/ZIF-8 membranes exhibit excellent thermal stability which present the high decomposition temperature of about 300 °C. The initial thermal weight loss of ZIF-8 powder and PEME-3:7/ZIF-8 membranes should be due to the decomposition of unreacted organic ligand 2-MIM and residual solvent in ZIF-8 particles. The thermal dimensional stability of PEME-3:7, PEME-3:7/ZIF-8(15%), and Celgard 2400 membranes were evaluated. As shown in Figure 4g, at 180 °C for 10 min, the Celgard 2400 is completely curled and deformed. When the temperature is further advanced to 250 °C, the Celgard 2400 has almost been pyrolyzed thoroughly. However, the PEME-3:7 and PEME-3:7/ZIF-8(15%) membranes still remain flat, showing much better thermal stability of PEME-3:7/ZIF-8(15%) than Celgard 2400. As shown in Figure S9, the DSC curve of ZIF-8 powder has no reaction peak in the range of 50–200 °C, indicating that it has excellent thermal stability in this temperature range. Moreover, with the increase of ZIF-8 growth, the Tg increased slightly, which is mainly due to the higher crystallinity of ZIF-8, resulting in an increase in the overall Tg.

### 2.3. Electrochemical Performance of ZIF-8/Epoxy Composite Electrolyte

**Figure 5:**
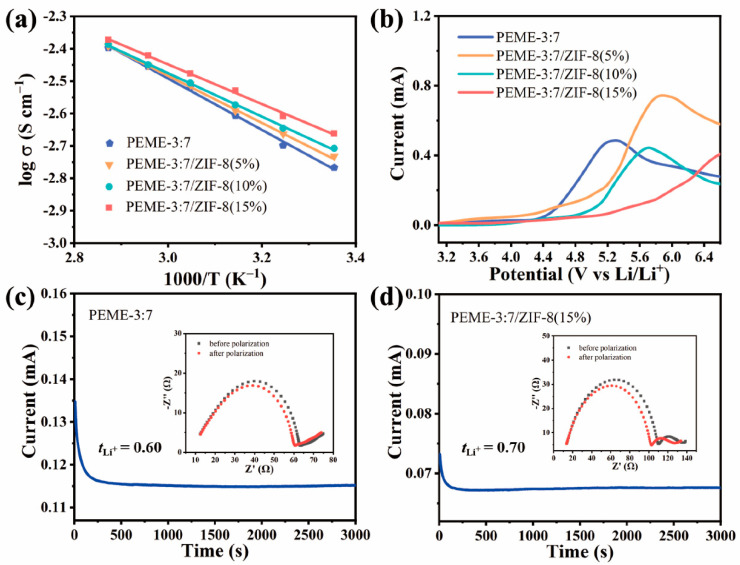
(**a**) Temperature dependence of ion conductivities of PEME-3:7 and PEME-3:7/ZIF-8 membranes. The solid lines are the Arrhenius fitting curves. (**b**) Linear scanning voltammograms of Li||PEME-3:7||SS and Li||PEME-3:7/ZIF-8||SS cells. The chronoamperometric profiles of (**c**) Li||PEME-3:7||Li and (**d**) Li||PEME-3:7/ZIF-8(15%)||Li cells with an applied potential difference of 10 mV (insets: the AC-impedance spectra of the cells before and after the polarization).

The effect of in situ grown ZIF-8 on the electrochemical performance of PEME-3:7/ZIF-8 was systematically investigated. The liquid uptake and ionic conductivities of PEME-3:7/ZIF-8 at room temperature can be obtained from Figure S10 using Equations (1) and (2), respectively, and the results are displayed in Table S2. Obviously, compared to PEME-3:7, all PEME-3:7/ZIF-8 show increased ionic conductivities, and the highest conductivity of 2.2 × 10^−3^ S cm^−1^ is obtained from PEME-3:7/ZIF-8(15%), which can be attributed to the strong nucleophilicity of ZIF-8 to facilitate the migration of Li ions and the increase in electrolyte uptake during the activation process. Figure 5a displays the temperature dependence of ionic conductivities for PEME-3:7 and PEME-3:7/ZIF-8(5~15%). The ion migration activation energies calculated according to the Arrhenius Equation (3) are 0.1572 eV, 0.1443 eV, 0.1331 eV, and 0.1215 eV, respectively. The activation energy of PEME-3:7/ZIF-8(15%) is the lowest, indicating that the migration of lithium ions in it is the easiest compared to the other samples. Moreover, as can be seen from Figure 5b, the oxidative degradation of the PEME-3:7 occurs at about 4.3 V (vs. Li^+^/Li), while the major anodic decomposition potential for PEME-3:7/ZIF-8 is about 5.0 V (vs. Li^+^/Li), implying enhanced electrochemical stability of the epoxy resin-based electrolyte by the addition of ZIF-8. The improved electrochemical stability window should be attributed to the effective capture ability of ZIF-8 for small molecules and the strong interaction between ZIF-8 and epoxy resin matrix. In addition to the higher ionic conductivity and wider electrochemical stability window of PEME-3:7/ZIF-8 than PEME-3:7, ZIF-8 with Lewis acid surface [40] has a strong interaction with PF_6_^−^, which might be beneficial to reduce the anion concentration polarization during charge/discharge and increase $t_{Li^{+}}$ [45]. To verify this, we obtained the Li-ion transference number ($t_{Li^{+}}$) of PEME-3:7 and PEME-3:7/ZIF-8 with the method of steady-state polarization and by Equation (4). As shown in Figure 5c,d and Figure S11, the $t_{Li^{+}}$ of PEME-3:7/ZIF-8(15%) is 0.70, which is higher than that of PEME-3:7 (0.60). The $t_{Li^{+}}$ of PEME-3:7/ZIF-8(5%) and PEME-3:7/ZIF-8(10%) are 0.64 and 0.68, respectively. This result is consistent with the former expectation. The increase in the $t_{Li^{+}}$ of PEME-3:7/ZIF-8(15%) can alleviate the polarization and delay the formation of Li dendrites, thereby enhancing the performance of LMBs.

**Figure 6:**
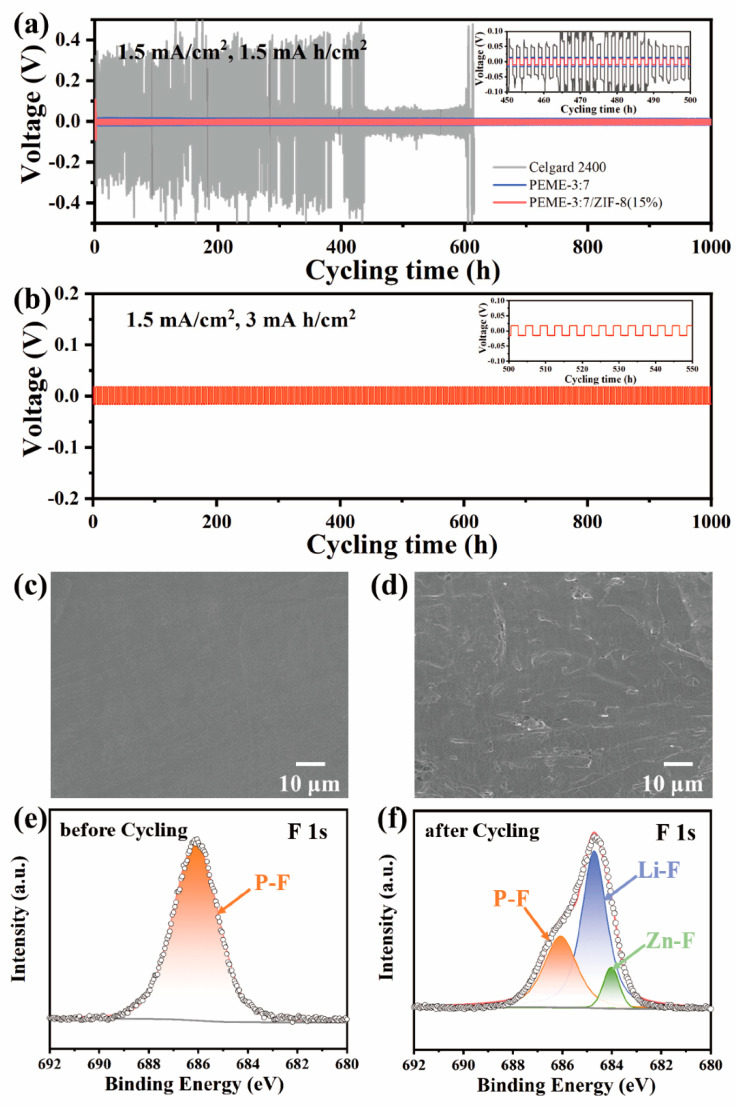
Voltage curves of (**a**) Li||Celgard 2400||Li, Li||PEME-3:7||Li, and Li||PEME-3:7/ZIF-8(15%)||Li at current density of 1.5 mA/cm^2^ for 1.5 mA h/cm^2^; (**b**) Li||PEME-3:7/ZIF-8(15%)||Li at current density of 1.5 mA/cm^2^ for 3 mA h/cm^2^. Surface SEM image of (**c**) pristine Li sheet; (**d**) Li anode of Li||PEME-3:7/ZIF-8(15%)||Li after 500 cycles at 1.5 mA/cm^2^ current density. XPS spectra of F 1s (**e**) before and (**f**) after PEME-3:7/ZIF-8(15%) membrane cycling.

To evaluate the dynamic stability of PEME-3:7/ZIF-8(15%) to the Li metal electrode, we assembled Li||PEME-3:7/ZIF-8(15%)||Li symmetric cells for constant current Li plating/stripping experiments at room temperature. For comparison, the symmetric cells of Li||Celgard 2400||Li with liquid electrolytes and Li||PEME-3:7||Li were also tested. Figure 6a displays the time-dependent voltage curves of the three cells at a current density of 1.5 mA cm^−2^, and each charge/discharge cycle was maintained for 2 h. For PEME-3:7/ZIF-8(15%), a relatively high voltage polarization could be observed during the initial few cycles, which might be related to the electrode activation and the formation of a stable SEI layer [11,46]. The overpotential then can be stabilized at about 10 mV over 1000 h, indicating a good electrode–electrolyte contact and stable lithium metal interface. The overpotential of Li||PEME-3:7||Li is about 15 mV, which is not much different from that of Li||PEME-3:7/ZIF-8(15%)||Li. In contrast, the symmetric cell containing Celgard 2400 has high overpotential under the same conditions with large fluctuations. The results show the excellent stability between PEME-3:7/ZIF-8(15%) electrolyte and lithium metal. As shown in Figure 6b and Figure S12, when the current areal capacity increases to 3 mA h cm^−2^ and 6 mA h cm^−2^ in each deposition/stripping cycle, PEME-3:7/ZIF-8(15%) can still maintain a stable, flat overpotential for over 1000 h and 800 h, respectively, indicating the excellent performance of PEME-3:7/ZIF-8(15%) in suppressing lithium dendrites. In addition to the unique pore structure and excellent mechanical properties of the PEME-3:7/ZIF-8(15%) electrolyte that can inhibit the growth of Li dendrites, two other factors play an important role in promoting uniform Li plating/stripping. One is that the high lithium-ion transference number of the PEME-3:7/ZIF-8(15%) electrolyte contributes to the uniform deposition of Li; another is that the N atom at one end in ZIF-8 contains a pair of lone electrons, which have strong nucleophilicity and electronegativity to stabilize lithium ions, thereby prolonging the nucleation time of lithium deposition and achieving uniform distribution of Li.

To further verify the abovementioned point of view, Figure 6c,d and Figure S13 show the surface SEM images of the fresh Li metal and after long-cycling Li metal electrode at 1.5 mA cm^−2^, respectively. It can be found that there is a smooth surface on the fresh Li metal. Meanwhile, the Li metal surface obtained from the cell using PEME-3:7/ZIF-8(15%) is much smoother than that of PEME-3:7 and Celgard 2400, without significant Li dendrites even after 1000 h, and the Li deposition was relatively dense. This further demonstrates that the PEME-3:7/ZIF-8(15%) can efficiently inhibit the formation of lithium dendrites. Furthermore, the XPS spectra of the PEME-3:7/ZIF-8(15%) membrane before and after cycling in the symmetric cells are obtained. The local XPS spectra of the F 1s element are shown in Figure 6e,f, the P-F bond in LiPF_6_ is at 686.1 eV before and after cycling, and the signal peaks correspond to ZnF_2_ and LiF at 684 eV and 684.7 eV after cycling, respectively. It can be concluded that an ionically conductive LiF and ZnF_2_-rich SEI layer is formed between the interfaces after cycling, which will greatly facilitate the charge transfer process, achieve uniform Li deposition, and a stable and long cycle life. Moreover, the LiF and ZnF_2_-rich SEI film can reduce the diffusion barrier of Li ions and improve the mechanical strength of the SEI layer. This not only helps to provide abundant Li ions replenishment and continuous transport pathways, but also inhibits the formation of Li dendrites [47].

**Figure 7:**
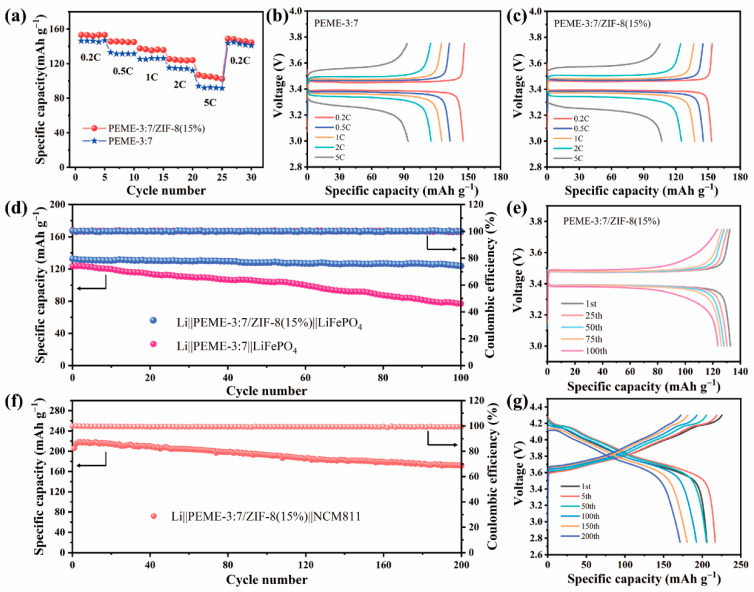
(**a**) Rate performances of the Li||LiFePO_4_ cells using PEME-3:7 and PEME-3:7/ZIF-8(15%). Charge–discharge curves of the (**b**) Li||PEME-3:7||LiFePO_4_ cell and (**c**) Li||PEME-3:7/ZIF-8(15%)||LiFePO_4_ cell at 0.2 C, 0.5 C, 1 C, 2 C, and 5 C. (**d**) Cycling performances of the Li||PEME-3:7||LiFePO_4_ and Li||PEME-3:7/ZIF-8(15%)||LiFePO_4_ cells at 0.5 C. (**e**) Voltage profiles of the Li||PEME-3:7/ZIF-8(15%)||LiFePO_4_ cell for different cycles at 0.5 C. (**f**) Cycling performances of the Li||PEME-3:7/ZIF-8(15%)||NCM811 cells at 0.2 C. (**g**) Voltage profiles of the Li||PEME-3:7/ZIF-8(15%)||NCM811 cell for different cycles at 0.2 C.

In order to investigate the practical application of the PEME-3:7/ZIF-8(15%) in LMBs, the electrochemical performance was evaluated on Li||LiFePO_4_ cells with a cathode loading of ≈2 mg cm^−2^ using PEME-3:7 and PEME-3:7/ZIF-8(15%) electrolyte and measured at room temperature. Figure 7a displays the rate performance of the Li||PEME-3:7||LiFePO_4_ and Li||PEME-3:7/ZIF-8(15%)||LiFePO_4_ cells. As can be seen, the Li||PEME-3:7/ZIF-8(15%)||LiFePO_4_ cell delivers discharge capacities of 153.2, 145.5, 137.5, 125.3, and 106.9 mA h g^−1^ at 0.2, 0.5, 1, 2, and 5 C (1 C = 170 mA h g^−1^), respectively, while the Li||PEME-3:7||LiFePO_4_ cell shows discharge capacities of 146.1, 132.9, 125.2, 115.3, and 94 mA h g^−1^. When the current density returns from 5 C to 0.2 C, the Li||PEME-3:7/ZIF-8(15%)||LiFePO_4_ and Li||PEME-3:7||LiFePO_4_ cells exhibit discharge capacities of 148.7 and 143.6 mA h g^−1^, respectively, indicating the better rate performance of the Li||PEME-3:7/ZIF-8(15%)||LiFePO_4_ cell than that of the Li||PEME-3:7||LiFePO_4_ cell. The corresponding charge/discharge curves of the Li||PEME-3:7||LiFePO_4_ and Li||PEME-3:7/ZIF-8(15%)||LiFePO_4_ cells at different rates are presented in Figure 7b,c, respectively. As can be seen, the Li||PEME-3:7/ZIF-8(15%)||LiFePO_4_ has lower polarization than the Li||PEME-3:7||LiFePO_4_ cell at 5 C. The results indicate that the rate performance of the cell using PEME-3:7/ZIF-8(15%) is much better than that of PEME-3:7, especially at high current rates, which can be ascribed to the growth of ZIF-8 to enhance the ionic conductivity, lithium ion migration, and electrochemical interfacial stability between the PEME-3:7/ZIF-8(15%) and lithium metal anode. Figure 7d displays the cycling performance of the Li||PEME-3:7||LiFePO_4_ and Li||PEME-3:7/ZIF-8(15%)||LiFePO_4_ cells for 100 cycles at a 0.5 C rate at room temperature. Clearly, the cell with PEME-3:7/ZIF-8(15%) shows a great coulombic efficiency and cycle stability, and its initial specific capacity is 132.8 mA h g^−1^. After 100 cycles, its capacity retention is 93.1% and coulombic efficiency is close to 100%. In contrast, the capacity of Li||PEME-3:7||LiFePO_4_ decreases dramatically during cycling, and its capacity retention is only 62% after 100 cycles. The results manifest that the Li||PEME-3:7/ZIF-8(15%)||LiFePO_4_ builds a robust and stable conductive interface during cycling, which accelerates the charge transfer process and achieves long cycle stability. As shown in Figure 7e, the Li||PEME-3:7/ZIF-8(15%)||LiFePO4 cell displays a significantly stable charge–discharge voltage plateau and has a small polarization voltage (0.08 V). To further verify the effectiveness and feasibility of PEME-3:7/ZIF-8(15%) in LMBs, full cells with LiNi_0.8_Mn_0.1_Co_0.1_O_2_ (NCM811) cathode (cathode loading ≈3 mg cm^−2^) were assembled and evaluated by charge–discharge cycling at 0.2 C between 2.75 and 4.3 V at room temperature. As shown in Figure 7f,g, the initial charging process exhibits a high electrode polarization, which might be caused by the inhomogeneous grain boundary contact of the NCM material in the initial state. After 5 cycles, the cycle reaches a steady state with a specific capacity of 216.8 mA h g^−1^. After 200 cycles, the specific capacity is 172.1 mA h g^−1^, which still maintains 83.2% of the initial specific capacity, proving that PEME-3:7/ZIF-8(15%) has great promise for application in LMBs.

## 3. Experimental Section

### 3.1. Materials

Epoxy resin E51 was purchased from Nanya Epoxy Resin Co., Ltd. (Kunshan, Jiangsu, China). Poly(propylene glycol)bis(2-aminopropyl ether) (D230) was purchased from Aladdin Biochemical Technology Co., Ltd. and 4,4′-diaminodicyclohexyl methane (PACM) was purchased from Macklin Biochemical Co., Ltd. (Shanghai, China). as curing agents. The polyethylene glycol 200 (PEG200, Mw = 200) and Poly(vinyl alcohol) (PVA 1799, degree of saponification: 98–99%) were purchased from J & K Scientific Ltd. Zn(CH_3_COO)_2_·2H_2_O was obtained from Energy Chemical; 2-methylimidazole (2-MIM) was obtained from Aladdin Biochemical Technology Co., Ltd. The electrolytes were formed by activation of 1M LiPF_6_ and EC/DMC (1:1, *v*/*v*) liquid electrolyte solution. Celgard 2400 were prepared by Celgard company. LiFePO_4_ for cathode preparation was acquired from Pulead Technology Industry Co. (Beijing, China).

### 3.2. Sample Preparation

Preparation of PVA-coated glass plates: Firstly, 1 g of PVA was added to 99 g of distilled water and stirred well at 100 °C to prepare an aqueous solution of PVA with a mass fraction of 1%. The prepared PVA aqueous solution was then poured onto clean glass plates, and the solution was spread evenly on the plate with a coating rod to form a water film, then the plates were heated on a hot table at 105 °C until the water was removed to obtain a glass plate coated with PVA.

Preparation of porous epoxy resin matrix electrolytes (PEME-x): Firstly, the epoxy resin E51 and the PEG200 (mass ratio 1:2.75) were stirred at 60 °C for 1 h until well mixed, then equal amounts of curing agent D230, D230, and PACM compound (where mass ratio of D230: PACM is 3:7, 5:5, and 7:3, respectively) were added, stirred well using the residual temperature, and then defoamed. The defoamed mixture was then poured onto a PVA-coated glass plate and another glass plate was placed on top to form a sandwich structure. Afterwards, the samples were heated at 80 °C for 2 h and then 120 °C for 2 h for curing. After curing, the samples were immersed in distilled water to remove the epoxy resin film and the PEG200 was removed by ultrasonically washing and soaking the epoxy resin film in distilled water. After that, the above epoxy resin film was dried in a freeze dryer for 12 h. Finally, the dried epoxy resin film was placed in a glove box and immersed in a liquid electrolyte of 1M LiPF_6_ with EC/DMC (1:1 by volume) for 24 h to obtain PEME-x. Corresponding to different ratios of D230 and PACM, the PEME-x were named PEME-3:7, PEME-5:5, PEME-7:3, and PEME-D230, respectively. The schematic diagram of the preparation procedure of the PEME-x membranes is illustrated in Figure 1a, and the formulations of the PEME-x series are listed in Table S1.

Preparation of ZIF-8/epoxy composite electrolytes (PEME-3:7/ZIF-8): Varying amounts of Zn(CH_3_COO)_2_·2H_2_O particles (5%, 10%, 15%, and 20% of the mass of epoxy E51) were added to the mixture of E51 and PEG200 before the addition of the curing agents (mass ratio of PACM:D230 is 3:7). The intermediate experimental steps were the same as described above. Afterwards, 2-methylimidazole (2-MIM) was dissolved in ethanol to obtain a clarified solution. The porous epoxy resin membranes prepared above with the addition of Zn(CH_3_COO)_2_·2H_2_O were placed into the 2-MIM solution and left for 24 h to allow the in situ growth of ZIF-8 on the PEME-3:7 membrane. The PEME-3:7/ZIF-8 membranes were then washed with ethanol and finally dried. The final experimental steps were the same as above. Depending on the Zn(CH_3_COO)_2_·2H_2_O content, the PEME-3:7/ZIF-8 membranes were named PEME-3:7/ZIF-8(5%), PEME-3:7/ZIF-8(10%), PEME-3:7/ZIF-8(15%), and PEME-3:7/ZIF-8(20%), respectively. The schematic diagram of the preparation procedure of the PEME-3:7/ZIF-8 membranes is illustrated in Figure 1b.

### 3.3. Characterization of the Materials

The surface and internal morphology of the materials were observed by scanning electron microscopy (SEM, JSM-7800F, JEOL, Tokyo, Japan). The structural characteristics and grain size of the prepared in situ grown ZIF-8 were analyzed by transmission electron microscopy (TEM, HT7700, JEOL, Tokyo, Japan). The composition of the functional groups of the materials were characterized by Fourier transform infrared spectroscopy (FTIR, Nicolet 8700, TMO, Waltham, MA, USA). The tensile properties of the materials were tested using a universal tensile machine (Instron-5567, Instron, Norwood, MA, USA). Brunauer Emmett Teller (BET) and High-performance Automatic Mercury Porosimetry (AutoPore Iv 9500, Micromertics Instrument Corp., Norcross, GA, USA) were used to determine the specific surface area and pore size distribution of the membranes. The crystal structure of ZIF-8 obtained in the materials were characterized by X-ray diffraction (XRD, Ultima IV, Rigaku Co., Tokyo, Japan) in the range of 3–90 °C. The chemical state of elements on the material surface was characterized by X-ray photoelectron spectroscopy (XPS, ESCALAB 250, TMO, Waltham, MA, USA).

The wetting behaviors of the membranes were characterized by OCA20 contact angle analyzer (Dataphysics, Germany). To characterize the thermal stability of the materials, thermogravimetric analysis (TGA, TMO, Waltham, MA, USA) was performed in the temperature range from room temperature to 800 °C at a rate of 10 °C/min under a nitrogen gas flow. The reactivity and Tg of each component were studied by Q20 differential scanning calorimeter (DSC, TMO, Waltham, MA, USA). The test temperature range was −80–300 °C at a heating rate of 10 °C/min.

To determine the electrolyte absorption (*η*) of the materials, the membranes were immersed in a liquid electrolyte for 24 h. *η* was determined using the following equation:

(1) $$\eta =\left(W_{1}-W_{0}\right)/W_{0}$$

where *W*_0_ is the weight of the initial membranes before aspiration and *W*_1_ is the weight of the electrolyte membranes after aspiration.

### 3.4. Electrochemical Characterization

Electrochemical impedance spectroscopy (EIS) of the symmetric cell SS||PEME||SS was carried out using an Autolab PGSTAT 302N with a frequency window from 1 MHz to 0.1 Hz at the corresponding test temperatures of 25 °C, 35 °C, 45 °C, 55 °C, 65 °C, 75 °C, and 85 °C, respectively. The ionic conductivity was calculated using the following equation:

(2) $$\sigma =\frac{L}{R_{b}\times S}$$

where *σ* is the ionic conductivity, *L* is the thickness of the electrolyte membranes, *R_b_* is the bulk resistance, and *S* is the contact area between the electrolyte and the electrode.

The ion migration activation energy of the electrolyte membranes was calculated from the Arrhenius Equation:

(3) $$\ln\sigma =\frac{-E_{a}}{RT}+\ln A$$

where *E_a_* is the ion migration activation energy, *R* is the molar gas constant, *T* is the test temperature, and *A* is the frequency factor (Arrhenius constant).

The electrochemical stability window of Li||PEME-3:7||SS and Li||PEME-3:7/ZIF-8||SS cells was investigated using linear sweep voltammetry (LSV). LSV tests were carried out at a scan rate of 10 mV s^−1^ over a voltage range from open circuit voltage to 8 V (vs. Li^+^/Li) at room temperature.

The lithium ion transference number ($t_{Li^{+}}$) in Li||PEME-3:7||Li and Li||PEME-3:7/ZIF-8||Li cells was measured using the steady-state polarization method at a polarization voltage of 10 mV (Δ*V*). The equation for $t_{Li^{+}}$ is as follows:

(4) $$t_{Li^{+}}=\frac{I^{s}\left(\Delta V-I^{0}R_{ct}^{0}\right)}{I^{0}\left(\Delta V-I^{s}R_{ct}^{s}\right)}$$

where *I^o^* and *I^s^* are the initial and steady-state current values, respectively, and $R_{ct}^{o}$ and $R_{ct}^{s}$ are the interfacial resistance between the electrolyte and the electrode measured before and after polarization, respectively.

The performance of the full cells was tested by assembling Li||LiFePO_4_ and Li||NCM811 cells using PEME-3:7 and PEME-3:7/ZIF-8 electrolytes. LiFePO_4_ cathodes were prepared by mixing LiFePO_4_ powder, conductive carbon black, and PVDF in the weight ratio of 8:1:1 with an appropriate amount of N-methyl-pyrrolidone (NMP) solvent to form slurry and using Al foil as the current collector. The obtained LiFePO_4_ cathodes were dried in a vacuum oven at 80 °C for 24 h. The cells were assembled in a glove box filled with argon gas. All the cells were tested on a battery tester (LAND CT2001A) for charge and discharge with a voltage range of 3–3.75 V at room temperature. NCM811 cathodes were prepared by mixing NCM811 powder, conductive carbon black, and PVDF in the weight ratio of 8:1:1 with an appropriate amount of N-methyl-pyrrolidone (NMP) solvent to form slurry and using carbonized Al foil as the current collector. The obtained NCM811 cathodes were dried in a vacuum oven at 110 °C for 24 h. The cells were assembled in a glove box filled with argon gas. All the cells were tested on a battery tester (LAND CT2001A) for charge and discharge with a voltage range of 2.75–4.3 V at room temperature.

## 4. Conclusions

In summary, a ZIF-8/epoxy composite electrolyte with a unique pore structure has been designed and successfully prepared by in situ growth of ZIF-8 in a porous epoxy matrix. It can not only significantly improve the mechanical strength to achieve uniform lithium deposition, but also effectively enhance the interface stability with lithium anode. The unique pore structure and the electronegativity of ZIF-8 with Lewis acid surface enable rapid lithium ion transport and restrict the movement of anions. Ultimately, the PEME-3:7/ZIF-8(15%) shows high ionic conductivity (2.2 × 10^−3^ S cm^−1^), wide electrochemical window (5 V), and a large Li^+^ transference number (0.70) at room temperature. Meanwhile, the formation of a fluoride-rich conductive SEI layer stabilizes the lithium-anode interface and enables uniform deposition of lithium ions, which can effectively suppress lithium dendrites and extend the cycle life. Even at a high current density of 1.5 mA cm^−2^ for 6 mA h cm^−2^, the Li-symmetric cell has a low and stable polarization voltage with a lifetime of more than 800 h. The Li||NCM811 cell using the PEME-3:7/ZIF-8(15%) exhibits high capacity and excellent cycling performance (83.2% capacity retention with 172.1 mA h g^−1^ capacity retained after 200 cycles at 0.2 C). The ZIF-8/epoxy composite electrolyte with unique pore structure will facilitate the practical application of highly stable and safe LMBs.

## Data Availability

The data presented in this study are available upon request from the corresponding author.

## Supplementary Materials

The following supporting information can be downloaded at: https://www.mdpi.com/article/10.3390/molecules27217488/s1, Figure S1: Surface and cross-sectional SEM images of (a,b) PEME-5:5, (c,d) PEME-7:3, (e,f) PEME-D230. Figure S2: N_2_ adsorption-desorption isotherm of (a) PEME-5:5 (b) PEME-7:3 (c) PEME-D230 (inset shows the pore size distribution of the membranes). (d) The pore size of different sample. Figure S3: Absorbance histogram for PEME-x with different curing agent ratios. Figure S4: The tensile strength and Young’s modulus histogram of PEME-x with different curing agent ratios. Figure S5: Surface and cross-sectional SEM images of (a,b) PEME-3:7/ZIF-8(5%), (c,d) PEME-3:7/ZIF-8(10%), (e,f) PEME-3:7/ZIF-8(20%). Figure S6: The (a–d) surface SEM images (e–h) surface Zn EDS mappings (i–l) cross-sectional SEM images (m–p) cross-sectional Zn EDS mappings of PEME-3:7/ZIF-8(5%), PEME-3:7/ZIF-8(10%), PEME-3:7/ZIF-8(15%), PEME-3:7/ZIF-8(20%) membranes. Figure S7: N_2_ adsorption-desorption isotherm of (a) PEME-3:7/ZIF-8(5%) (b) PEME-3:7/ZIF-8(10%) (c) PEME-3:7/ZIF-8(15%) (inset: the pore size distribution of the membrane). (d) Pore size distribution of PEME-3:7/ZIF-8(15%) membrane under mercury intrusion test (inset: the mercury intrusion-evolution curve of the membrane). Figure S8: The flexibility measurement of PEME-3:7/ZIF-8(15%) membrane (a) before liquid electrolyte immersion, (b) after liquid electrolyte immersion. Figure S9: DSC test curves of different components. Figure S10: (a) Impedance spectrum of SS//PEME-3:7/ZIF-8(0-15%)//SS cells at room temperature. (b) absorbance histogram for PEME-3:7/ZIF-8(0-15%). Figure S11: (a) Li//PEME-3:7/ZIF-8(5%)//Li and (b) Li//PEME-3:7/ZIF-8(10%)//Li cells with an applied potential difference of 10 mV (insets: the AC-impedance spectra of the cells before and after the polarization). Figure S12: Voltage curves of Li//PEME-3:7/ZIF-8(15%)//Li at current density of 1.5 mA/cm^2^ for 6 mA h/cm^2^. Figure S13: Surface SEM image of (a) Li anode of Li||Celgard 2400||Li (b) Li anode of Li||PEME-3:7||Li after 500 cycles at 1.5 mA/cm^2^ current density. Table S1: The formulations of the porous epoxy resin membranes. Table S2: The ionic conductivity at room temperature and electrolyte uptake of PEME-3:7/ZIF-8(0–15%).

## Sample Availability

Not available.
