# Supplementary material for: Novel In Situ Growth of ZIF-8 in Porous Epoxy Matrix for Mechanically Robust Composite Electrolyte of High-Performance, Long-Life Lithium Metal Batteries
Source: Molecules. 2022 Nov 3;27(21):7488. doi: 10.3390/molecules27217488 (PMC9654070; doi:10.3390/molecules27217488)
Supplement: Supplementary file 1 [file molecules-27-07488-s001.zip › molecules-1986736-supplementary.pdf]

Supporting information

Novel in situ Growth of ZIF-8 in Porous Epoxy Matrix for Mechanically Robust Composite Electrolyte of High-Performance, Long Life Lithium Metal Batteries

Wenjie Zhang[1], Jianlin Long[1], Haijun Wang[2], Jinle Lan[1*], Yunhua Yu[1*], and Xiaoping Yang[1,3]

1. State Key Laboratory of Organic-Inorganic Composites, College of Materials Science and Engineering, Beijing University of Chemical Technology, North Third Ring Road 15, Chaoyang District, Beijing 100029, P. R. China
2. State Key Laboratory of Fluorinated Functional Membrane Materials, Zibo, Shandong 256401, P. R. China
3. Foshan (Southern China) Institute for New Materials, Foshan 528200, Guangdong, P.R. China

*Corresponding author: lanjl@mail.buct.edu.cn ; yuyh@mail.buct.edu.cn

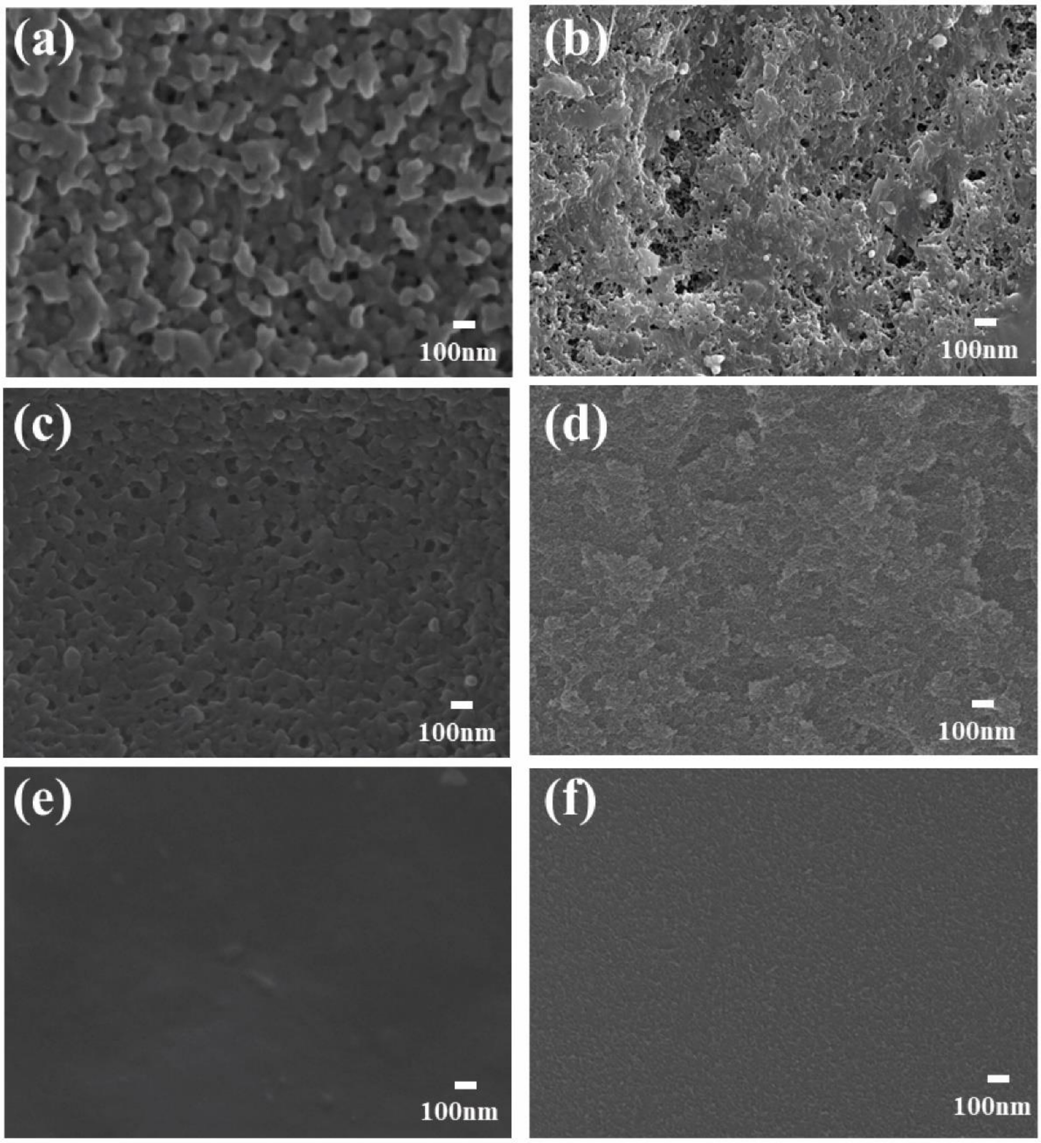

Figure S1. Surface and cross-sectional SEM images of (a-b) PEME-5:5, (c-d) PEME-7:3, (e-f) PEME-D230.

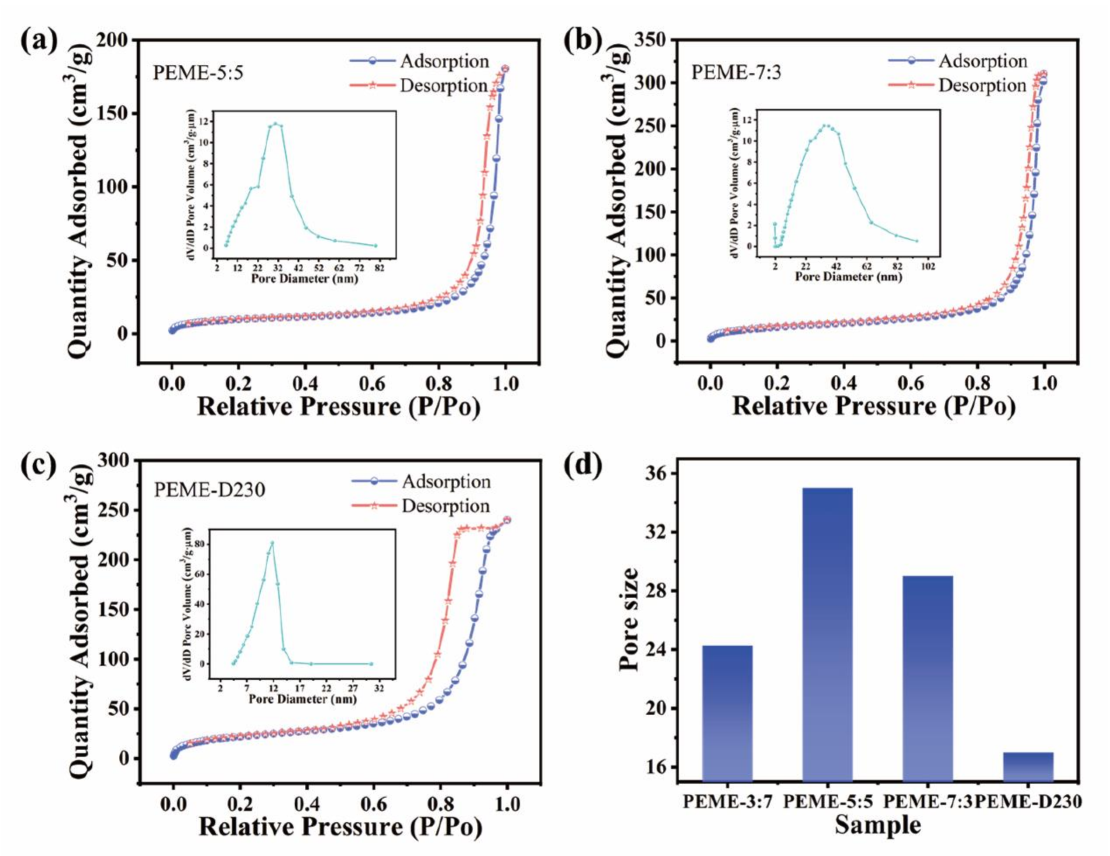

Figure S2. N_2 adsorption-desorption isotherm of (a) PEME-5:5 (b) PEME-7:3 (c) PEME-D230 (inset shows the pore size distribution of the membranes). (d) The pore size of different sample.

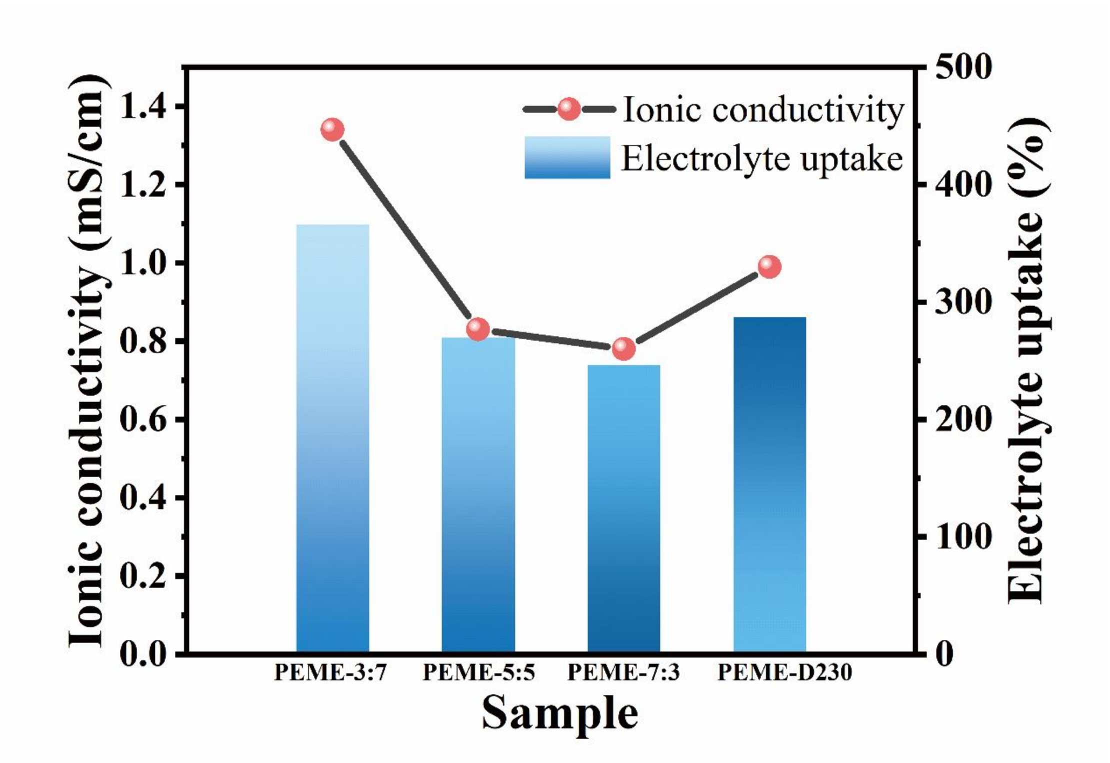

Figure S3. Absorbance histogram for PEME-x with different curing agent ratios.

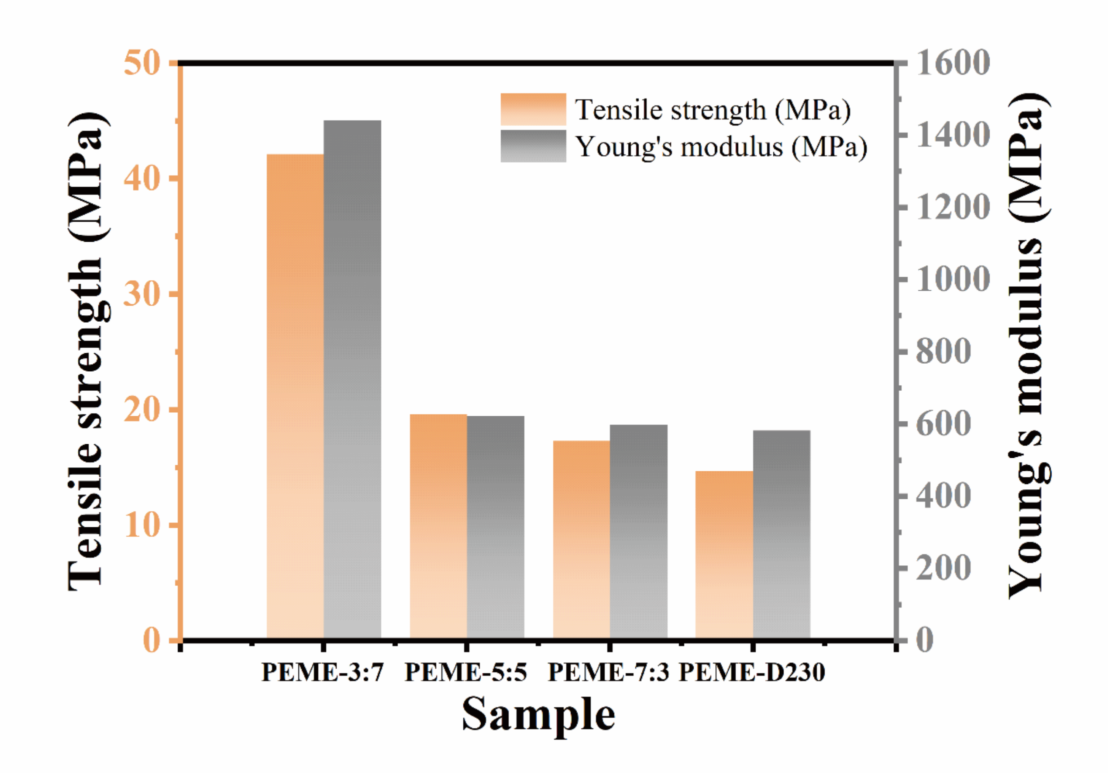

Figure S4. The tensile strength and Young's modulus histogram of PEME-x with different curing agent ratios.

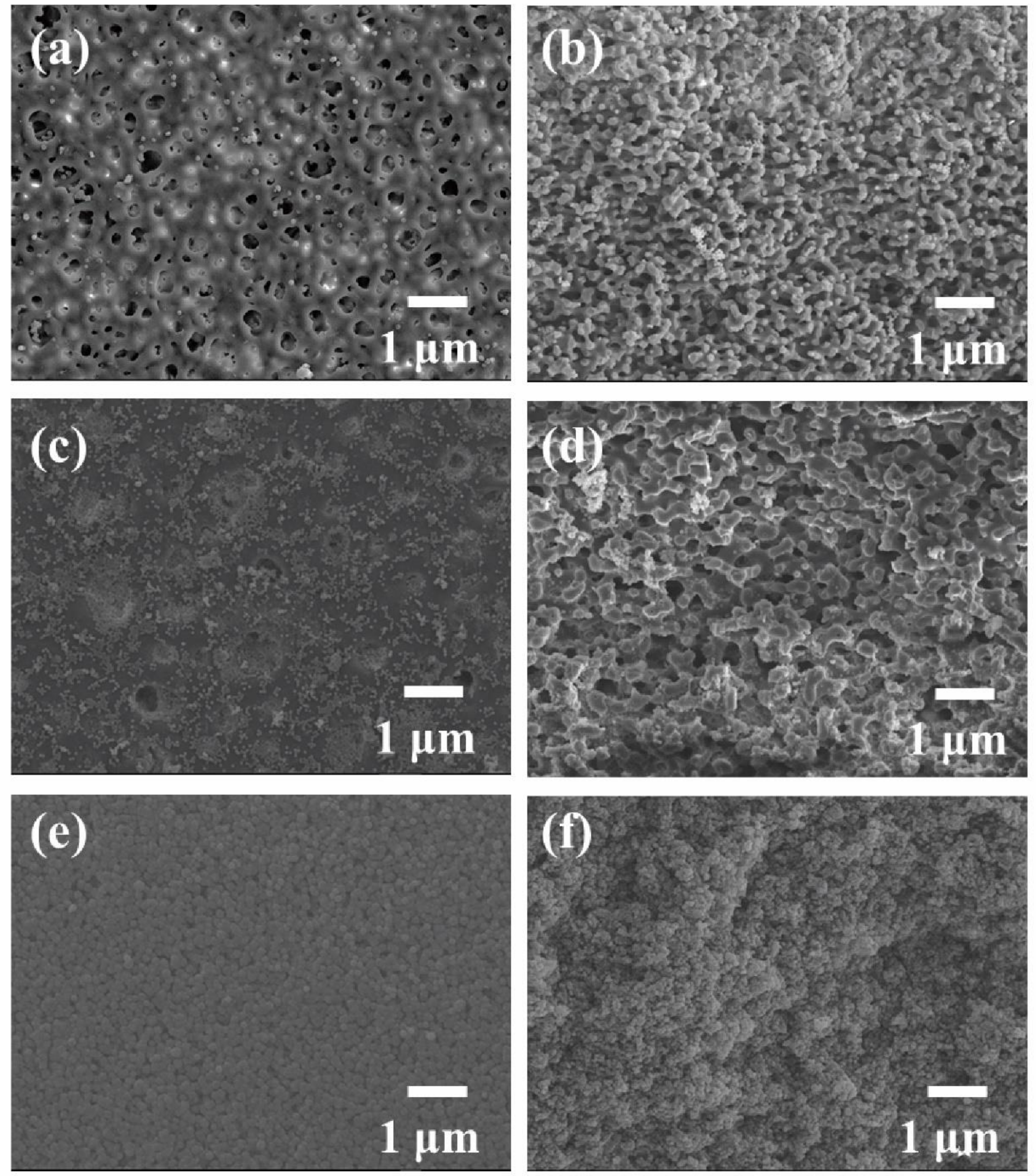

Figure S5. Surface and cross-sectional SEM images of (a-b) PEME-3:7/ZIF-8(5%), (c-d) PEME-3:7/ZIF-8(10%), (e-f) PEME-3:7/ZIF-8(20%).

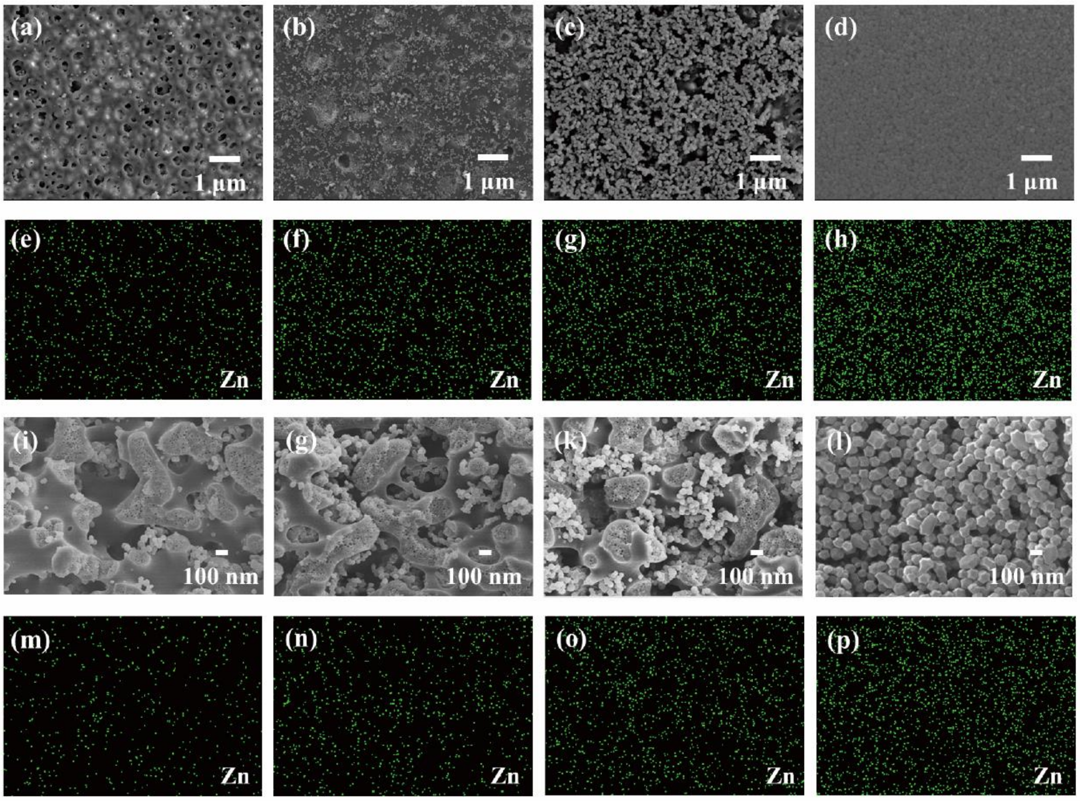

Figure S6. The (a-d) surface SEM images (e-h) surface Zn EDS mappings (i-l) cross-sectional SEM images (m-p) cross-sectional Zn EDS mappings of PEME-3:7/ZIF-8(5%), PEME-3:7/ZIF-8(10%), PEME-3:7/ZIF-8(15%), PEME-3:7/ZIF-8(20%) membranes.

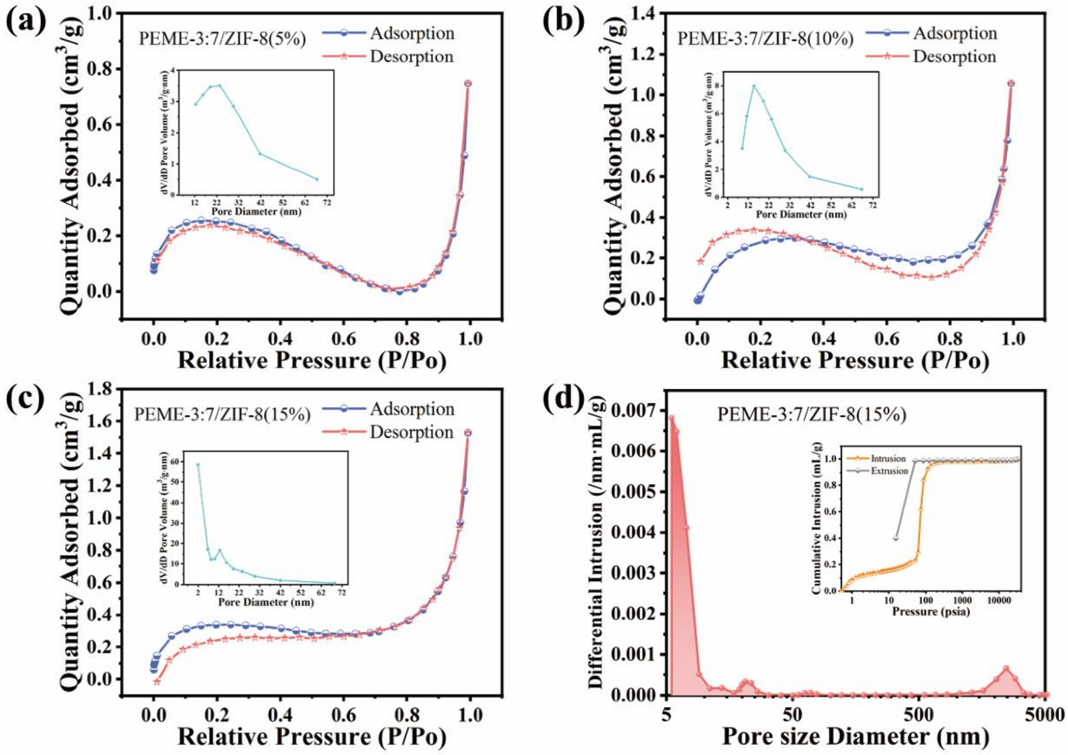

Figure S7. N_2 adsorption-desorption isotherm of (a) PEME-3:7/ZIF-8(5%) (b) PEME-3:7/ZIF-8(10%) (c) PEME-3:7/ZIF-8(15%) (inset: the pore size distribution of the membrane). (d) Pore size distribution of PEME-3:7/ZIF-8(15%) membrane under mercury intrusion test (inset: the mercury intrusion-evolution curve of the membrane).

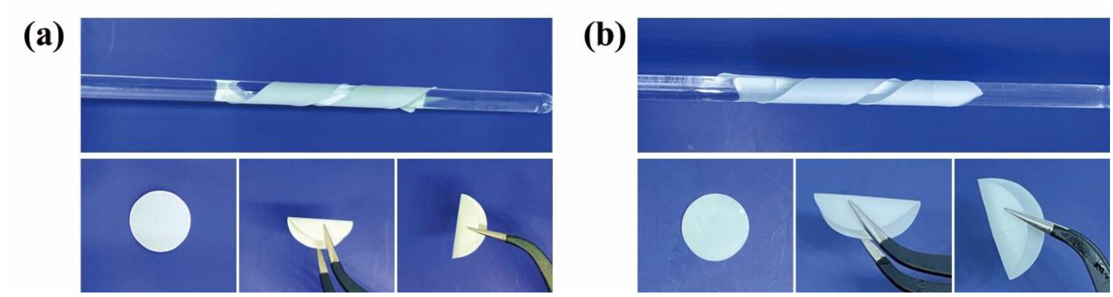

Figure S8. The flexibility measurement of PEME-3:7/ZIF-8(15%) membrane (a) before liquid electrolyte immersion, (b) after liquid electrolyte immersion.

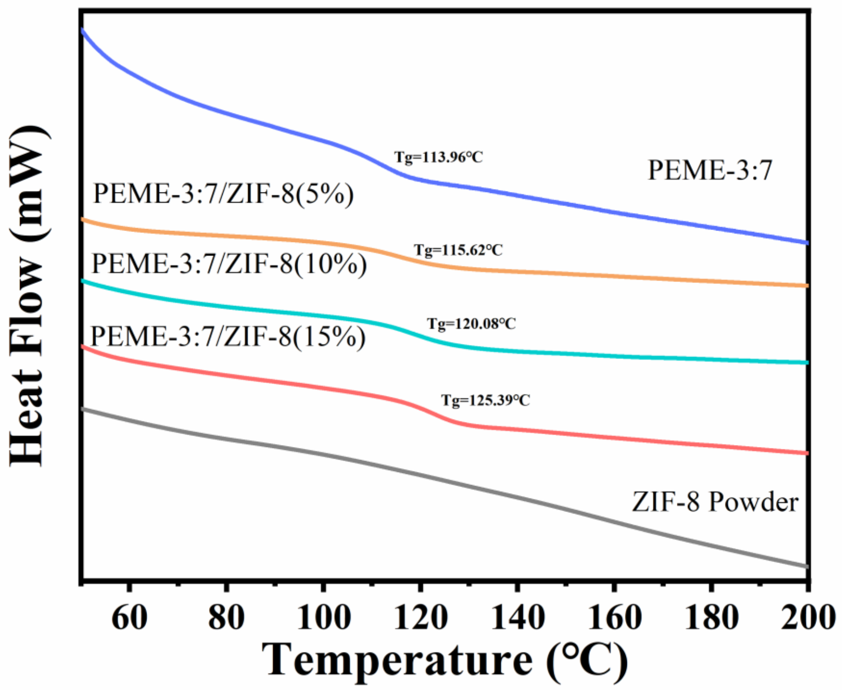

Figure S9. DSC test curves of different components.

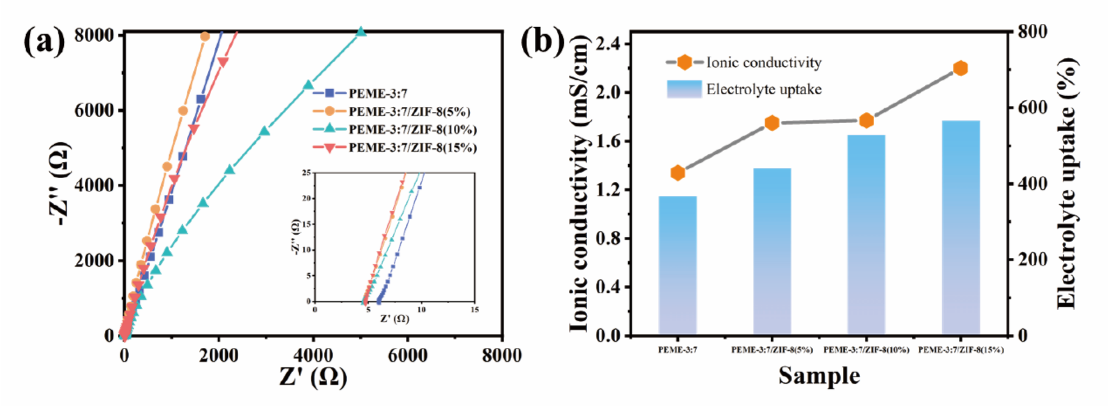

Figure S10. (a) Impedance spectrum of SS//PEME-3:7/ZIF-8(0-15%)//SS cells at room temperature. (b) absorbance histogram for PEME-3:7/ZIF-8(0-15%).

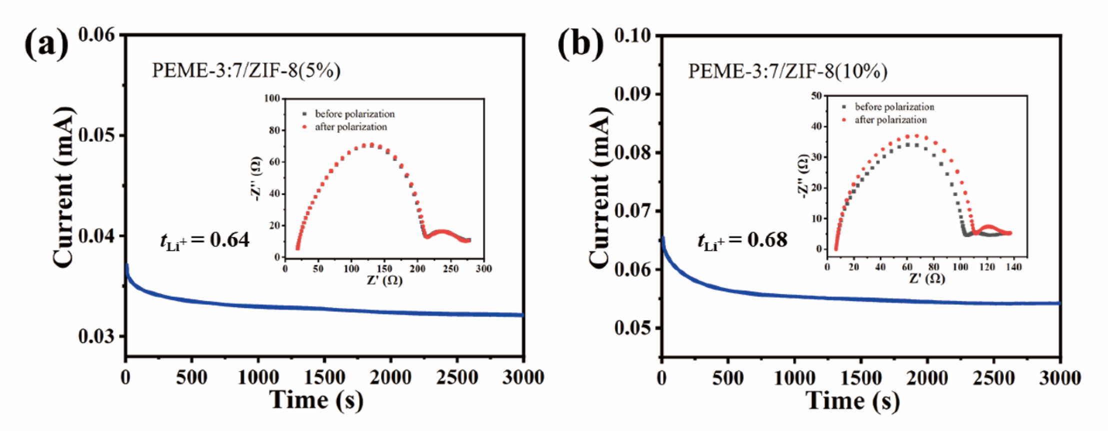

Figure S11. (a) Li//PEME-3:7/ZIF-8(5%)//Li and (d) Li//PEME-3:7/ZIF-8(10%)//Li cells with an applied potential difference of 10 mV (insets: the AC-impedance spectra of the cells before and after the polarization).

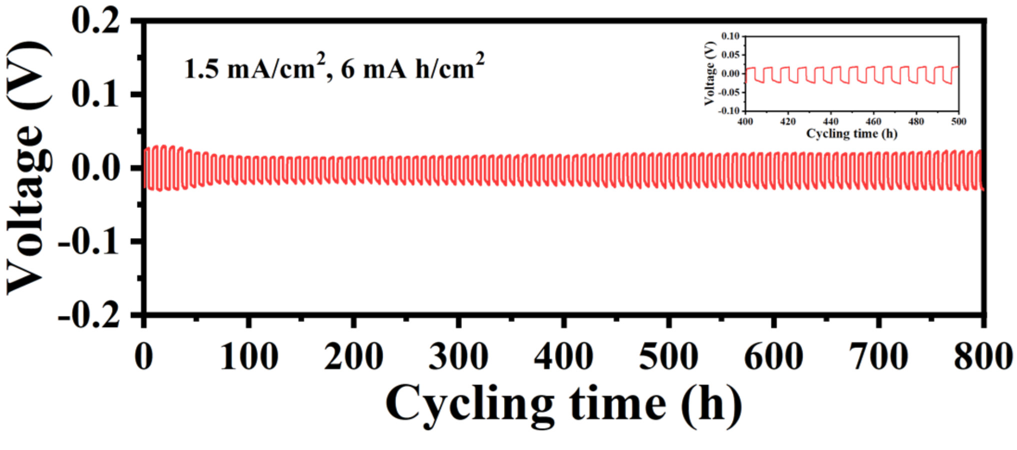

Figure S12. Voltage curves of Li//PEME-3:7/ZIF-8(15%)//Li at current density of 1.5 mA/cm^2 for 6 mA h/cm^2.

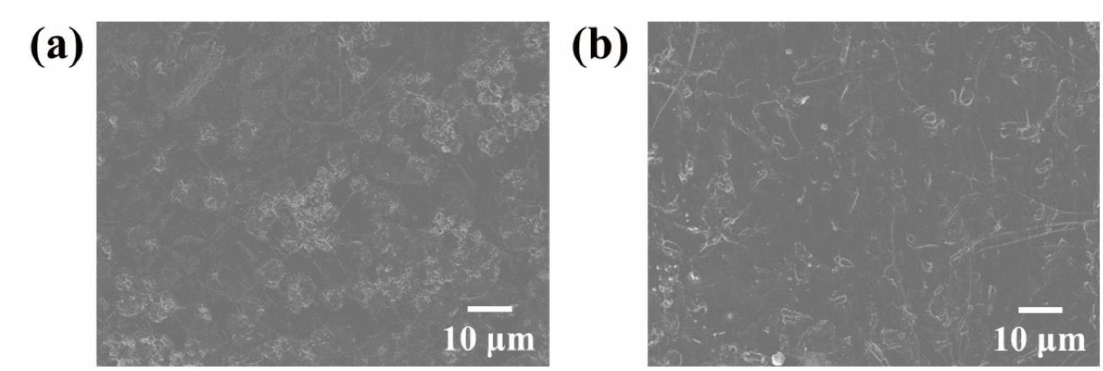

Figure S13. Surface SEM image of (a) Li anode of Li||Celgard 2400||Li (b) Li anode of Li||PEME-3:7||Li after 500 cycles at 1.5 mA/cm^2 current density.

Table S1. The formulations of the porous epoxy resin membranes.

Samples	DGEBA (g)	PEG200 (g)	D230 (g)	PACM (g)
PEME-3:7	1	2.75	0.088	0.188
PEME-5:5	1	2.75	0.146	0.134
PEME-7:3	1	2.75	0.205	0.080
PEME-D230	1	2.75	0.293	0

Table S2. The ionic conductivity at room temperature and electrolyte uptake of PEME-3:7/ZIF-8(0-15%).

Samples	bulk resistance (Ω)	ionic conductivity (S/cm)	electrolyte absorption (%)
PEME-3:7	5.97	1.34×10^{-3}	366.1
PEME-3:7/ZIF-8(5%)	4.65	1.75×10^{-3}	439.7
PEME-3:7/ZIF-8(10%)	4.5	1.77×10^{-3}	528.9
PEME-3:7/ZIF-8(15%)	4.75	2.2×10^{-3}	566.7
